# Enhanced Clinical Decision-Making and Delivery of Minimally Invasive Care Using the ICCMS4D Integrated with Hands-Free Fluorescence-Based Loupes and a Chemomechanical Caries Removal Agent

**DOI:** 10.1055/s-0043-1764423

**Published:** 2023-04-27

**Authors:** Gal Hiltch, Liviu Steier, José Antonio Poli de Figueiredo

**Affiliations:** 1 Department of Dentistry, Federal University of Rio Grande do Sul—UFRGS, Porto Alegre, Brazil; 2 Department of Preventive and Restorative Sciences, Dental School, University of Pennsylvania- UPENN, Philadelphia, Pennsylvania, United States; 3 Department of Morphological Sciences, Federal University of Rio Grande do Sul–UFRGS, Porto Allegre, Brazil

**Keywords:** caries detection, caries diagnostics, fluorescence, ICDAS

## Abstract

The purpose of this case report was to evaluate an approach for enhanced clinical decision-making while providing minimally invasive treatment, using the combined International Caries Classification and Management System (ICCMS 4D) with hands-free fluorescence-enhanced loupes (Reveal, Designs for Vision Inc., New York City, NY, United States) and a chemo-mechanical caries removal agent (Papacárie Duo Gel, Formula & Acao, Sao Paulo, Brazil). In recent decades, a shift towards a conservative approach to caries management has developed. The use of adequate operative techniques and correct decision-making are of paramount importance for early caries detection and tooth-preserving operative care. The use of noninvasive fluorescence-based adjuncts for enhanced diagnostic accuracy has gained popularity. Fluorescence describes the absorption of photons by a molecule, followed by its partial emission at a longer wavelength. Fluorescence detection of bacterial activity is largely based on the red/orange fluorescence emanating from bacterial metabolism, and has been shown to be found particularly in active caries and mature anaerobic plaque. The combined approach of using the standardized visual International Caries Detection and Assessment System (ICDAS) with fluorescence as an operative adjunct was shown to enhance the advantages of both systems. The approach may potentially increase detection sensitivity without compromising the specificity of the visual tactile method. A recent hands-free device is aimed to combine simultaneous diagnosis and therapy (theragnosis) using fluorescence, while overcoming possible obstacles to workflow continuity. The “Reveal” fluorescence-enhanced loupes are fitted with a dual white light/fluorescence light, and are said to allow the clinician to conduct any operational procedure with optional fluorescence guidance, from detection to caries removal, to treatment completion. Using the described method, a comprehensive pathway that emphasizes evidence-based information, correct clinical decision-making, and a minimally invasive therapeutic approach was proposed. The approach may represent a potential advancement in providing minimally invasive caries care.

## Introduction

Traditionally, carious lesions were detected at late stages, and their management was primarily based on operative procedures. In the last decades, a better understanding of the pathophysiologic process of caries progression, along with the developments of ion-releasing materials (IRB), has led to contemporary methods for their management. 1 2 3 4 5 6 7

In 2005, an “International Caries Detection and Assessment System” (ICDAS) was developed to standardize the variations in which caries lesions were detected, assessed, and classified among the dental disciplines. 5 Active lesions were described as yellowish/light brown, and soft/leathery, while arrested lesions were defined as darkly stained and hard textured. Built on the ICDAS system, the “International Caries Classification and Management System” (ICCMS) was developed. To put the ICCMS into practice, a concise version (ICCMS 4D) was published in 2019, integrating risk-based minimally invasive and preventive caries management. 6

Current recommendations on deep caries management support the removal of caries tissue up to sound tissue at its periphery to allow for a quality adhesive seal. 1 2 Centrally, removal up to firm/leathery affected dentine is preferred, since the affected dentin is low in bacterial load and capable of re-mineralizing with a variety of IRB (e.g., calcium silicate based, high-viscosity glass ionomer cements [GICs], and silver diamine fluoride). 3 4

The development of minimally invasive caries approaches has led to various supplemental devices, based on fluorescence technologies. These devices have been shown to aid early lesion detection, especially in approximal regions. 8 9 10 Moreover, the systems only use light and do not expose the patients to harmful ionizing radiation. Many of the available devices are based on quantitative light-induced fluorescence (QLF) technology, which employs light to the tooth structure in the violet-blue spectrum. 11 Under these conditions, sound, incipient, and infected tissue emit different light. Sound tooth structure fluoresces green. 12 Demineralized regions appear darker due to increased light scattering 11 and bacterially infected regions appear bright red/orange. 12 The red/orange fluorescence is assumed to originate mainly from porphyrin derivatives within the dental biofilm, and especially protoporphyrin IX. 12 13 14 Several studies have shown that the combination of ICDAS/QLF can potentially increase detection sensitivity without compromising the high specificity of the visual methods (i.e., correctly identifying the diseased region while avoiding overtreatment of healthy regions). 14 15 16 17

**Fig. 1:**
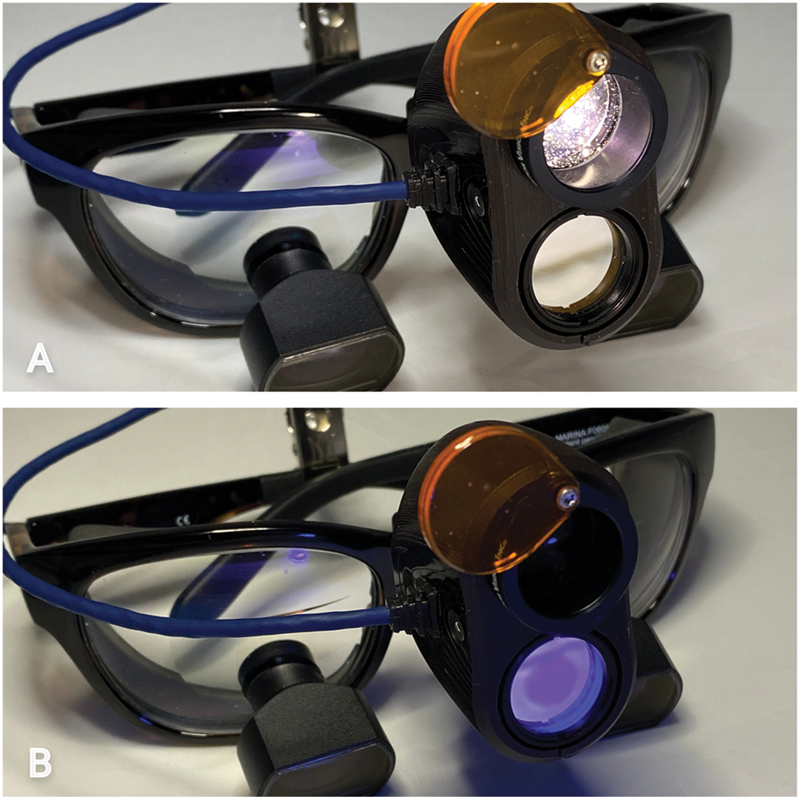
“Reveal” fluorescence-enhanced-magnification-loupes equipped with a ( **A** ) dual white light and ( **B** ) fluorescence 405-nm light.

Currently, fluorescence-based diagnostic devices can be classified into handheld light-emitting diode lamps (LED), handheld camera-integrated devices, and hands-free devices. 8 9 10 As handheld devices may occupy space and require operative dexterity, some devices have incorporated fluorescence capabilities into magnification loupes to facilitate operational procedures. The Reveal fluorescence-enhanced magnification loupes system (Designs for Vision, Inc., NY, United States; Fig. 1 ) is one of them. The loupes are fitted with filtered telescopic lenses, and dual daylight/fluorescence 405-nm light LEDs. The setup is said to allow the clinician to conduct any operational procedure with fluorescence guidance, from diagnosis to treatment completion. 10 18

The aim of the current report was to evaluate a combined use of hands-free fluorescence-enhanced theragnosis (FET) with the “Reveal” loupes, integrated into ICCMS 4D, as an approach to empower the clinician toward enhanced clinical decision-making and providing minimally invasive treatment. The minimally invasive operative technique for infected caries removal was used with the Papacárie Duo Gel (Formula & Acao, Sao Paulo, Brazil), a chemomechanical caries removal agent (CMCR). 19

## Case Report

The care protocol describes a comprehensive pathway that emphasizes evidence-based information gathering, correct clinical decision-making, and a minimally invasive therapeutic approach. With the informed consent of the patient, the photos of the case were documented, using Nikon Z-6 (Nikon Corporation, Yokohama, Japan), fitted with the “Reveal” filter and LED. The outlined protocol is based on the key principles of the ICCMS 4D system integrated with FET:

1. Determining patient-level caries risk, according to the caries management by risk assessment (CAMBRA)-adopted ICCMS guidelines.
2. Detection and assessment of caries stages and activity with a dual-light examination. A dual-visual examination using white light/fluorescence light is performed on the teeth following the ICDAS examination protocol. Caries activity is confirmed with fluorescence examination by the presence of red fluorescence activity on the surface of clean teeth.
3. Decision-making to form a diagnosis based on caries classification (initial, moderate, extensive) and patient-level risk.
4. *Management:* Nonoperative care (e.g., fluoride varnish, silver diamine fluoride, resin sealants) or tooth-preserving operative care under FET and minimally invasive techniques are performed (e.g., sono-abrasives, CMCR, photoactive disinfection, hand instruments). Prevention and control interventions are established.

## Clinical Case

A 22-year-old man presented to the clinic for a consultation regarding his front teeth. The patient lacked any contributing factors to health problems. The patient reported a vague dental history regarding previous dental treatments. Following the initial assessment, the patient was classified with a high caries risk. The patient made it clear that at the initial stage he is only interested in treating his front teeth.

**Fig. 2:**
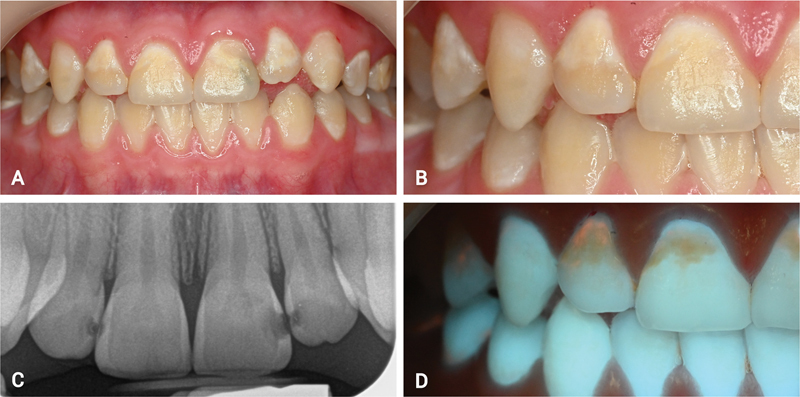
Initial pictures of the case. ( **A** ) Frontal view under white light. ( **B** ) High-magnification frontal view under white light. ( **C** ) Radiograph showing the lesions on the front teeth. ( **D** ) High-magnification frontal view under fluorescence light.

Caries classification was performed under dual white light/fluorescence light with the “Reveal” fluorescence-enhanced loupes. All buccal aspects of the teeth were classified as active ICDAS 1 or 2 ( Fig. 2A ), displaying a rough surface and whitish/yellowish opaque color. Under fluorescence light, dark lesions of demineralized enamel were observed. The lesions were covered by a thin uncleansable red fluorescence ( Fig. 2D ), which seemed embedded to the tooth structure (could not be removed by a dental probe or a prophy paste).

**Fig. 3:**
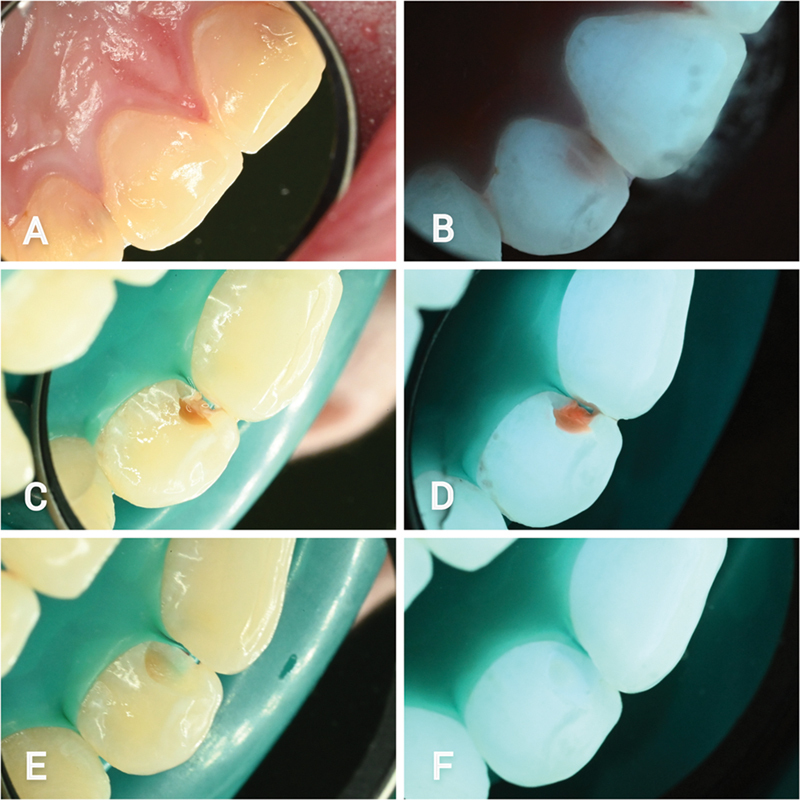
Palatal aspect of the right upper lateral incisor. ( **A** ) White light initial lesion. ( **B** ) Fluorescence light initial lesion. ( **C** ) Cavity opening under white light. ( **D** ) Cavity opening under fluorescence light. ( **E** ) White light view following selective removal of infected tissue. ( **F** ) Fluorescence view following selective removal of infected tissue.

**Fig. 4:**
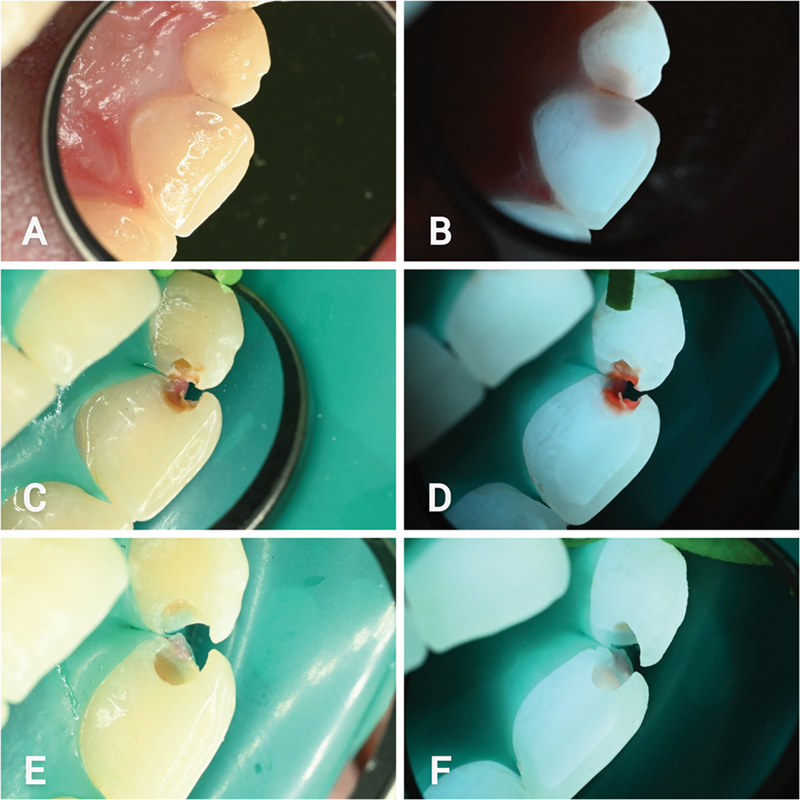
Palatal aspects of the upper left central and lateral incisors. ( **A** ) White light initial lesion. ( **B** ) Fluorescence light initial lesion. ( **C** ) Cavity opening under white light. ( **D** ) Cavity opening under fluorescence light. ( **E** ) White light view following selective removal of infected tissue. ( **F** ) Fluorescence view following selective removal of infected tissue.

A deep blue shadow (ICDAS 4) was observed under the buccal surface of the left maxillary central incisor. White light examination of the palatal aspect of the anterior maxillary teeth revealed distinct shadows on the mesial aspect of the right upper lateral incisor ( Fig. 3A ) and on the distal and mesial surfaces of the upper left central and lateral incisors ( Fig. 4A ). Under fluorescence light, shiny brown lesions were identified ( Figs. 3B and 4B ). A radiographic assessment further confirmed the three lesions as moderate, roughly reaching the middle third of dentin. An additional incipient lesion reaching approximately half of the enamel was observed on the mesial aspect of the right central incisor.

According to the ICCMS system, clinical assessment of the lesion severity merges the ICDAS classification with radiographic assessment into initial, moderate, or extensive stages. The three main lesions were assessed as moderate active, and an initial active stage was established for the buccal surfaces of the teeth.

Nonoperative care was decided on the initial active white lesions using a professional fluoride varnish (Bifluoride 10, VOCO GmbH, Cuxhaven, German). Additionally, the patient was scheduled for a professional cleaning and hygiene instruction. The importance of correct oral hygiene habits and limited free sugar intake was stressed. The patient was already using a high fluoride toothpaste (1,450 ppm), so an additional fluoride mouth rinse was recommended. Recall within a 3-month interval was scheduled. It was agreed that prior to the restorative phase of the white lesions, proper maintenance should be accomplished to ensure a good outcome from the treatment.

A tooth-preserving operative care regimen was agreed for the three moderate active lesions. A cavity opening was made using a sono-abrasive handpiece (SONICflex 2003L, KaVo dental GmbH, Biberach, Germany). As the cavity was opened under fluorescence guidance with the Reveal system, a shiny red autofluorescence was gradually revealed from within the lesions, until the full extent of the infected tissue was observed ( Figs. 3D and 4D ).

**Fig. 5:**
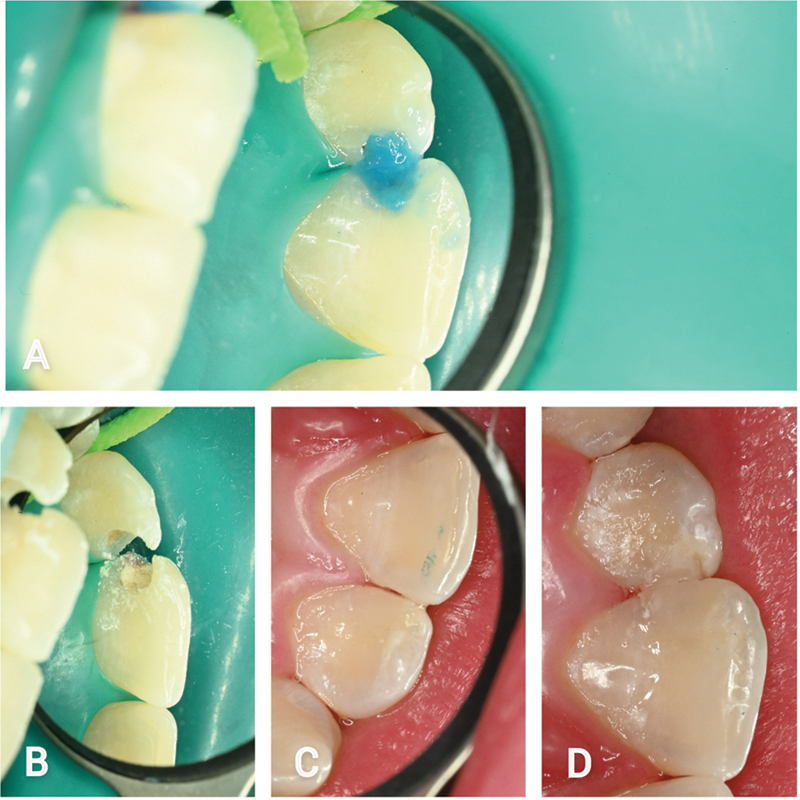
Excavation and reconstruction procedure. ( **A** ) Selective removal of infected tissue with Papacárie Duo Gel, according to manufacturer instructions. ( **B** ) IRB application to the affected regions. ( **C** ) Composite reconstruction of the right upper lateral incisor. ( **D** ) Composite reconstruction of the upper left central and lateral incisors.

For selective removal of infected tissue, Papacárie Duo Gel, a CMCR, was used ( Fig. 5A ). Under white light/fluorescence light treatment guidance, the infected tissue from within the lesions was excavated up to a firm dentin to the touch ( Figs. 3E and 4E ). Under fluorescence light, healthy tissue, completely free from red fluorescence, was found at the periphery of the lesions. Centrally, a faint pink/orangey fluorescence dentin remained, firm to the touch ( Figs. 3F and 4F ).

The reconstruction procedure was performed accordingly: selective enamel etch for 20 seconds and dentin for 15 seconds with 37% phosphoric acid, 15 seconds of rinsing with air/water spray, followed by air drying for 5 seconds, and a 30-second application of chlorhexidine (CHX) 2% to inactivate matrix metalloproteinases. Application of an IRB to the affected regions (Harvard MTA Universal, Harvard Dental international GmbH, Hoppegarten, Germany) while allowing a 5-minute setting time until the next clinical procedure ( Fig. 5B ). The adhesion procedure was performed according to manufacturer recommendations with OptiBond FL (Kerr Corporation, Orange, United States), followed by a composite reconstruction with GrandioSO nanohybrid composite (VOCO GmbH, Cuxhaven, Germany; Fig. 5C , 5 ). The polishing procedure was performed with the Dimanto (VOCO GmbH, Cuxhaven, Germany) single-step diamond polisher.

## Discussion

Early detection of a caries lesion and its activity assessment has a great impact on clinical decision-making. Incipient and noncavitated active lesions are preferably managed nonoperatively. Moderate and extensive active lesions may need tooth-preserving operative treatment, and treatment of the inactive lesions mainly focuses on aesthetic improvement. Before deciding on the treatment protocol, clinicians first need to establish a correct diagnosis based on data gathered in the clinical examination.

Traditional methods for caries identification showed low accuracy and reproducibility. 6 To improve its shortcomings, an enhanced visual system was developed: the ICDAS. The ICDAS has been shown to be accurate and reproducible. 14 15 16 17 Visual examination, however, tends to have high specificity and low sensitivity. 17 To improve the detection of caries lesions, the use of adjunct tools, such as fluorescence-based devices, is encouraged. In recent years, several systematic reviews and meta-analyses have been conducted on the efficacy of such devices. 20 21 While studies showed controversial results with their effectiveness as stand-alone devices, the combined approach of ICDAS and fluorescence seems to enhance the benefits from both systems.

To date, most of the studies on fluorescence-based diagnostic tools were related to handheld devices. Some devices may occupy space and require operative dexterity, which may disturb the clinical workflow. The “Reveal” fluorescence-enhanced loupes system was recently introduced as an adjunct for clinical practice, which aimed to minimize some of these disadvantages. Coated with transparent filters and a dual white light/fluorescence light, the loupes add the possibility to conduct any operational procedure with fluorescence guidance, from diagnosis to treatment completion. The proposed approach in this study integrated these advantages with each step of the well-established ICCMS 4D treatment protocol for enhanced caries prevention and management.

During the classification and activity assessment stage, the use of fluorescence light was perceived to be helpful. When the 405-nm light was applied to the teeth, the demineralized regions appeared dark, owing to increased light scattering by the porous surface ( Fig. 2D ). Additionally, the lesions were covered by a thin uncleansable plaque of red fluorescence, indicating bacterial activity and colonization ( Fig. 2D ). Studies on red autofluorescence have shown it is particularly found among active caries lesions, as well as from mature plaque and its calcified form (calculus). 12 13 14 15 The red fluorescence is assumed to originate from porphyrin derivatives in the biofilm. The emanating fluorescence is assumed to result from a complex interspecies interaction rather than the presence of a single species. 10 Under fluorescence light, the detection of three moderate lesions on the palatal aspects of the affected teeth was substantially easier to recognize, as bright brown color emitted from within the teeth ( Figs. 3B and 4B ). The description in the report supports the quantitative study by Steier et al, 18 which showed improved visualization and monitoring of dentinal lesions with the “Reveal” fluorescence-enhanced loupes.

The treatments of the caries lesions in the report were tailored according to their location, classification, and radiological extent. Initial active lesions were treated with a fluoride varnish, as it was shown to re-mineralize the enamel and arrest active lesions. 3

A tooth-preserving operative care regimen was selected for the moderate-active lesions, as their shape and extent were rendered inaccessible for nonoperative care.

The extent of caries tissue removal is centered on dentin hardness as the main criteria (soft/leathery/firm/hard). However, as the level of hardness is based on qualitative parameters, differentiating between the layers and their boundaries poses some challenges. Various methods are encouraged for careful caries removal. Among them are excavation with hand instruments, polymer burs, air abrasion, sono-abrasion, and CMCR. 1 7 22 In the case report, a sono-abrasive handpiece was used (SONICflex 2003L) for cavity opening and a CMCR (Papacárie Duo Gel) for selective caries removal. Sonic handpieces provide great tactile feedback. While hard tissues are easily removed, soft tissues are not, making overpreparation highly unlikely. Additionally, they produce good surface finishing, lack of enamel cracks, and a good seal for bonding. 7 To aid in the selective removal of infected tissue, Papacárie Duo Gel, a CMCR, was used. While not the most time-efficient tools, CMCRs have the unique property to act on the partially degraded collagen within the infected dentin, allowing its removal by a blunt instrument, and preserving the demineralized affected dentin. 19 Its usage, which does not require rotary tools or anesthesia, has gained high acceptance in pediatric dentistry and among patients suffering from dental anxiety.

The infected tissue was excavated up to the firm dentin with white light/fluorescence light treatment guidance. The soft heavily infected outer dentin showed intense red fluorescence. The firm affected dentin was faintly pink/orangey. A study by Trippe et al correlated the tactile assessment of soft and leathery dentin with red and pink fluorescence, respectively. 23 However, the reverse correlation was found to be inconclusive, since some hard samples showed pink or red fluorescence. While hard dentin is perceived as “sterile,” bacteria are still present in all dentin layers, as supported by the bacterial microbiome analysis. 23

**Fig. 6:**
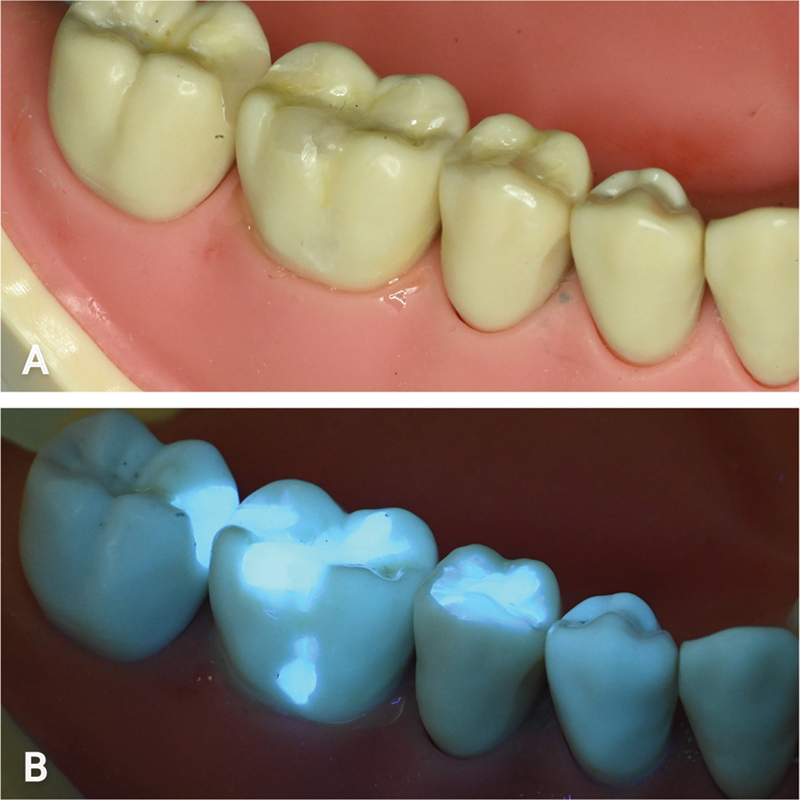
Fluorescence identification of restorative materials on a typodont model. ( **A** ) White light view. ( **B** ) Fluorescence light view.

Treatment completion was confirmed by the absence of restorative materials on unwanted surfaces under fluorescence light. Fluorescence-inducing pigments are added to dental composites and resin cement to improve their aesthetics under daylight. Ultraviolet (UV) exposure, especially in unaged composites, results in intense blue fluorescence, facilitating their discovery ( Fig. 6 ). The use of fluorescence identification of restorative materials is well documented in splint removal, orthodontic brackets debonding, and dental forensics. 24 The use of a clean, instantaneous, hands-free adjunct may be a welcoming addition for clinical armamentarium. From potential aid in lesion discovery, to caries tissue removal, to monitoring in the restorative phase. Further high-quality quantitative and qualitative studies are needed to support the conclusions of the report.

## Conclusion

ICMMS 4D integrated with FET with the Reveal loupes may provide a clean, instantaneous, and noninvasive visual adjunct, which is potentially an advancement in providing minimally invasive caries management.
